# Antiangiogenic effects of pazopanib in xenograft hepatocellular carcinoma models: evaluation by quantitative contrast-enhanced ultrasonography

**DOI:** 10.1186/1471-2407-11-28

**Published:** 2011-01-20

**Authors:** Xiao-Dong Zhu, Ju-Bo Zhang, Pei-Li Fan, Yu-Quan Xiong, Peng-Yuan Zhuang, Wei Zhang, Hua-Xiang Xu, Dong-Mei Gao, Ling-Qun Kong, Lu Wang, Wei-Zhong Wu, Zhao-You Tang, Hong Ding, Hui-Chuan Sun

**Affiliations:** 1 Liver Cancer Institute and Zhongshan Hospital, Fudan University, Shanghai 200032, People's Republic of China; 2 Department of Ultrasound, Zhongshan Hospital, Fudan University, Shanghai 200032, People's Republic of China

## Abstract

**Background:**

Antiangiogenesis is a promising therapy for advanced hepatocellular carcinoma (HCC), but the effects are difficult to be evaluated. Pazopanib (GW786034B) is a pan-vascular endothelial growth factor receptor inhibitor, the antitumor effects or antiangiogenic effects haven't been investigated in HCC.

**Methods:**

In vitro direct effects of pazopanib on human HCC cell lines and endothelial cells were evaluated. In vivo antitumor effects were evaluated in three xenograft nude mice models. In the subcutaneous HCCLM3 model, intratumoral blood perfusion was detected by contrast-enhanced ultrasonography (CEUS), and serial quantitative parameters were profiled from the time-intensity curves of ultrasonograms.

**Results:**

In vitro proliferation of various HCC cell lines were not inhibited by pazopanib. Pazopanib inhibited migration and invasion and induced apoptosis significantly in two HCC cell lines, HCCLM3 and PLC/PRF/5. Proliferation, migration, and tubule formation of human umbilical vein endothelial cells were inhibited by pazopanib in a dose-dependent manner. In vivo tumor growth was significantly inhibited by pazopanib in HCCLM3, HepG2, and PLC/PRF/5 xenograft models. Various intratumoral perfusion parameters changed over time, and the signal intensity was significantly impaired in the treated tumors before the treatment efficacy on tumor size could be observed. Mean transit time of the contrast media in hotspot areas of the tumors was reversely correlated with intratumoral microvessel density.

**Conclusions:**

Antitumor effects of pazopanib in HCC xenografts may owe to its antiangiogenic effects, and the in vivo antiangiogenic effects could be evaluated by quantitative CEUS.

## Background

Hepatocellular carcinoma (HCC), one of the most vascularized solid cancers [1], is the sixth most common cancer and the third most common cause of death from cancer worldwide [2]. This disease is usually diagnosed at an advanced stage, and therefore it presents a dismal prognosis, owing to the lack of effective systemic treatment options and the underlying liver disease [3]. Overall survival may be improved after the clinical application of sorafenib or other molecular targeting agents [4,5]. Tumor angiogenesis plays a critical role in the development and progression of HCC, which suggests that antiangiogenic therapy is effective in inhibiting tumor progression. The role of vascular endothelial growth factor (VEGF) and VEGF receptors (VEGFR) in the angiogenesis and progression of HCC has been well documented [6-8], providing a rationale for investigating VEGF or VEGFR-targeted therapies. Several clinical studies support antiangiogenic therapies in HCC, including bevacizumab [9,10], sorafenib [5], and others [11]. With the advent of these antiangiogenic therapies, there is increased interest in determining the clinical effects of these agents. Given the novel mechanism of action of these targeted agents, the classical definition of tumor response, tumor size reduction, may need to be revised. Thus there is a need for better understanding of how imaging methods may be utilized to assess the physiologic status of the tumor neovasculature and the activity of these agents in clinical use.

The change of intratumoral microvessel density (MVD) has been widely used to detect the response of antiangiogenic therapy in animal models, but its predictive value of treatment response was questioned in clinical settings because of some limitations[12,13]. For example, repeated biopsy to facilitate histological examination is usually impossible in clinical settings, especially in patients with HCC, which is often associated with coagulation dysfunction or ascites. Therefore noninvasive or minimally invasive methods is needed to monitor the biological response. Tumor perfusion measured by imaging methods, such as dynamic contrast-enhanced computed tomography (DCE-CT) and dynamic contrast-enhanced magnetic resonance imaging (DCE-MRI), have been reported [14-17]. With the use of microbubble contrast agents, contrast-enhanced ultrasonography (CEUS) could also provide a sensitive method to detect tumor perfusion [18-21]. With more convenient application, the diagnostic value of CEUS is similar to DCE-CT and DCE-MRI [22,23]. CEUS is nowadays widely used in diagnosis [24] or in predicting the treatment efficacy of local therapies [25-27] in HCC, but assessment of the efficacy of the targeted therapies was scarcely reported. CEUS, combined with a computerized imaging analysis system, can depict the perfusion with serial parameters to quantitative blood perfusion in multiple characteristics by analysing the time-intensity curve (TIC) obtained from regions of interest (ROI). In two previous studies assessing angiogenesis[20] or efficacy of antiangiogenic therapy[19] with CEUS, the correlation between MVD and parameters of CEUS was controversial [19,20], and only a few parameters were quantified, while the dynamic changes of the parameters during antiangiogenic therapy were not well observed.

In this study, the antitumor effect of pazopanib (GW786034B; GlaxoSmithKline, Collegeville, PA), a small-molecule tyrosine kinase inhibitor of VEGFRs, platelet-derived growth factor receptors, and c-Kit tyrosine kinases [28], were evaluated in 3 human HCC xenograft models. By analysis of dynamic images of CEUS, intratumoral blood perfusion was profiled by serial quantitative parameters, and biologic implications of these parameters were evaluated. Some CEUS parameters may be used as surrogate markers of MVD or used to quantitatively measure the intratumoral perfusion changes in the context of antiangiogenic treatment.

## Methods

### Cell lines and cell culture

Human HCC cell lines with high metastatic capacity (HCCLM3, MHCC97-H, and MHCC97-L, which were established by our institute [29]) or low metastatic capacity (SMCC7721, HepG2, and PLC/PRF/5) from ATCC cell bank were grown as a monolayer culture in Dulbecco's modified Eagle's medium or RPMI-1640 supplemented with 10% fetal calf serum, 100 U/mL penicillin, and 50 μg/mL streptomycin. Human umbilical vein endothelial cells (HUVEC, ScienCell Research Laboratories, San Diego, CA) and tumor-derived endothelial cells (TEC), established as previously described [30], were maintained on an endothelial cell medium (ScienCell Research Laboratories), and cells at passage 3 or 4 were chosen for experiments. All these cells were cultured at 37°C in a 5% CO_2_, 95% air environment in humidified incubators. Pazopanib, provided by GlaxoSmithKline in a purified powder, were reconstituted in dimethyl sulphoxide (DMSO) at a concentration of 300 μg/mL before use.

### Proliferation assay and Apoptosis assays

CellTiter-Glo^® ^luminescent cell viability assay (Promega, Madison, WI) was used to test the proliferation of cells in response to different concentrations of pazopanib, following the manufacturer's instructions. Briefly, 2 × 10^3 ^cells, HUVEC, TEC, SMCC7721, HCCLM3, MHCC97-H, MHCC97-L, HepG2, and PLC/PRF/5 were plated in the 96-well opaque-walled multiwell plates in culture medium. Control wells containing medium without cells were set to obtain a value for background luminescence. After adherent culture, pazopanib (final concentrations of 0, 1, 2, 5, 10, and 20 μg/mL) was added to each well and incubated for 72 h, and 80 μL CellTiter-Glo reagent was then added in each well. After being mixed and incubated at room temperature for 10 min, the luminescence of each well was recorded. HCCLM3, HepG2, and PLC/PRF/5 cells which were cultured in serum-free DMEM with 25 μg/mL pazopanib or vehicle for 6 h were collected and analysed for the presence of apoptotic cells using Annexin V-FITC Apoptosis Detection Kit (BD Pharmingen, San Jose, CA) following the manufacturer's instruction. Flow cytometry analysis was performed using FACS Calibur cytometer (R&D Systems, Minneapolis, MN). Replicate assays were done.

### Tumor cell migration and invasion assay

Transwell chamber inserts (Corning Inc, Corning, NY) with filter membrane pore size of 8 μm were coated with 80 μL Matrigel (0.8 mg/mL, BD Bioscience, Mountain View, CA). Tumor cells, HCCLM3, HepG2, and PLC/PRF/5, at the concentration of 5 × 10^5^/mL serum-free DMEM were incubated with 25 μg/mL pazopanib or vehicle at the upper chamber. DMEM containing 10% FBS was added to the lower compartment. Seventy-two hours later, cells that migrated through the permeable membrane were fixed in paraformaldehyde, stained with Giemsa, and counted under an inverted light microscope at 100× magnification. Each assay was done at least in triplicate. Migration assay were applied similarly without coating the upper chamber with Matrigel, and the cells migrated through the membrane were counted at 48 h.

### Wound-healing assay

HUVEC were seeded in 24-well plates and incubated for 12 h; the monolayer-adherent HUVEC were established. A line was scratched by a sterilized pin to wipe off the adherent cells in this line to create a wound. After being washed with PBS, pazopanib was then added to the medium (final concentrations of 0, 1, 4, 20, and 100 μg/mL) and a control was set up with an equal concentration of DMSO. Seven hours later, the migration of HUVEC was assessed using an inverted light microscope at 50× magnification; the distances HUVEC migrated in the 7 h were measured.

### In vitro angiogenesis assay (tubule formation assay)

In vitro formation of capillary-like structures was studied on growth factor-reduced Matrigel (BD Biosciences) diluted 1:1 on ice with cold endothelial cell medium in a 96-well plate. 5 × 10^4 ^HUVEC were inoculated on the surface of the Matrigel, after the adherent culture under normoxic conditions was reached and pazopanib (final concentrations of 0, 10, 20, and 40 μg/mL) was added. Tubule formation of HUVEC was assessed 3 and 6 h later under an inverted light microscope at 50× magnification.

### Animals

Male BALB/c nu/nu mice (4-6 wk old) of around 20 g (Shanghai Institute of Materia Medica, Chinese Academy of Sciences, Shanghai) were housed in laminar-flow cabinets under specific pathogen-free conditions. The mice were cared for and handled according to the U.S. Public Health Service Policy on Humane Care and Use of Laboratory Animals. The experimental protocol was approved by the Shanghai Medical Experimental Animal Care Committee. Mice were inoculated subcutaneously in the right dorsal part near the lower pole of right kidney with 5 × 10^6 ^HCCLM3, which could induced high incidence of lung metastasis when implanted either subcutaneously or orthotopically in nude mice [29], or PLC/PRF/5 cells with low metastatic capacity. Orthotopic HepG2 xenograft model was established by implantation of tumor blocks at the average volume of 1 mm^3^. Two weeks later, the xenograft model was established which was confirmed by conventional ultrasound with a high-frequency transducer; mice were then randomized into a pazopanib-treated group and a control group in a size-matched manner. Pazopanib was suspended in 0.5% hydroxypropylmethyl cellulose (Sigma-Aldrich, St. Louis, MO) and 0.1% Tween-80 (Sigma-Aldrich) in water as a vehicle (pH 1.3-1.5) and was given 40 mg/kg daily by oral gavage. The control group received the vehicle alone at the same schedule and route of administration. Tumor burden was measured every week by ultrasound when CEUS was applied for subcutaneous HCCLM3 tumor model. Animals were sacrificed to obtain the tumors for histological study at the indicated time points. To evaluate the therapeutic effects of pazopanib, a pilot study was performed at a higher dosage (60 mg/kg/d) in HCCLM3 xenograft model in advance (Additional file 1, Figure S1).

### CEUS protocols

The sonography investigators were blinded in regard to the groups. Animals of the subcutaneous HCCLM3 model were imaged on days 0, 7, 14, and 21 after treatment. Conventional ultrasound and harmonic CEUS were performed using a commercially available ultrasound system iU22 (Philips, Bothell, WA). A L17-9 linear array transducer was used for the confirmation of planted tumor and the measurement of tumor volume, and a L9-3 linear array wideband transducer combined with Pulse Inversion Harmonic technique was used for CEUS study. Imaging settings, including mechanical index (0.1), frame rate (18 Hz), compress (36 dB), gain, focus, depth, and depth gain compensation line, were established after optimization at the beginning and were fixed in this study. Mice were anesthetized with sodium pentobarbital (50 mg/kg intraperitoneally) and placed in the prone position on a polystyrene plastic pad. The centrifuged ultrasound coupling gel was filled to the thickness of 5 mm for maintenance between the surface of the transducer and the skin of the tumor. To avoid the potential functional effects on the endothelial cells of the antiangiogenic agents [12], CEUS was performed at least 8 h (range, 8 - 12) after the daily administration of pazopanib. At each imaging session, tumor volumes were assessed by conventional B-mode imaging and calculated as 0.52 × length × width × depth. During the contrast session, the longitudinal scan was chosen to display the largest dimension of the tumor and the right kidney. The transducer was then held in this position through the examination. Animals received a bolus injection of 0.02 mL (45 μg/mL) microbubble contrast agent (SonoVue, Bracco SpA, Milan, Italy) within 1-2 s. The contrast medium consists of a suspension of sulphurhexafluoride microbubbles surrounded by a thin layer of phospholipid and palmitic acid that allows the microbubbles to withstand several passes through the pulmonary capillaries. Each bolus injection of SonoVue was through a 27-gauge needle mounted to the tail vein, followed by a 0.2 mL saline flush. To control the quality of imaging, the enhancement in the imaging plane of the cortex of the ipsilateral kidney was also observed simultaneously. All the images were recorded in digital cine clips from the beginning of injection; recordings lasted for at least 2 min and were transferred in Digital Imaging and Communications in Medicine format to a PC for further analysis.

### Imaging data analysis

Dynamic digital data sequences of CEUS were analysed off-line with the software SW-UCS (Shenwei Imaging Technology Company, Shanghai, China) by the sonography investigators. After browsing the dynamic perfusion of the whole tumor frame by frame, two ROIs were placed to generate two initial time-intensity curves (TICs) presenting dynamic changes of signal intensity in the selected region. One ROI was placed to cover the tumor area as a whole, and the other one was located in the most enhanced area in the tumor on CEUS, which was referred as a "hotspot" area. The shape of ROI was a circle drawn manually and the area of ROI approximately ranged from 5 mm^2 ^to 800 mm^2^. By tracking every frame of contrast-enhanced images, the average intensity and initial TIC of each ROI were obtained. The initial TIC of each ROI was analysed and fitted with a least mean square method adaptable to bolus injection [31,32] and a series of quantitative parameters were calculated automatically with a mathematical function $I(t)=a_{0}+\begin{array}{cc} a_{1} & \frac{e^{-a_{2}t}}{1+e^{-a_{3}(t-t_{0})}} \end{array}$, which was adaptable to the kinetics of bolus injection as reported by Postert, et al. [32] Serial quantitative parameters of each ROI achieved from fitted TIC solved at every pixel in each ROI were calculated automatically (Additional file 1, Figure S2). The obtained TIC typically presented a baseline phase prior to the contrast and then a rapid increase in the signal intensity to a peak level followed by a slow washout phase. Quantitative parameters derived from the fitted curve (Table 1), including increased signal intensity from baseline (ISI), and rate of signal increase (RSI) in the rising period, rate of washout of 50% contrast material (RWO), mean transit time (MTT) of the contrast material, area under curve (AUC), and blood flow coefficient (BF; AUC/MTT × 2). These parameters could be classified into two types-volume related parameters, including AUC, ISI, and BF, and velocity related parameters, including RSI, RWO, and MTT. The volume related parameters were used to define the volume of blood perfusion in the ROI, while the velocity related parameters were used to define change of blood flow within a unit time in the area of interest. To reduce the variability among different subjects, which was probably due to the potential variation in the amounts of contrast entering the circulation, the volume-related parameters were standardized by dividing the corresponding parameters detected in the ipsilateral kidney and were presented as ratios.

**Table 1:** Terms used to portray the perfusion by contrast-enhanced ultrasonography

Acronym	Parameter	Notes
TIC	time-intensity curve	A curve to describe the signal intensity of contrast media in a region of interest changes over time when a series of ultrasonograms are analysed by specific software offline
ROI	region of interest	A circle drawn in the interested region for generating the time-intensity curve. In this study, one ROI took the tumor region as a whole, the other one, "hotspot", was located in the highest enhanced area on contrast-enhanced ultrasonography, which were determined by the two sonography investigators
ISI	increased signal intensity	The difference between baseline to the maximum increase in signal intensity in a region of interest
RSI	rate of signal increase	The rate of signal enhancement from baseline to initial peak in a region of interest
RWO	rate of washout of 50% of contrast material	The rate of signal intensity reduced to 50% of initial peak of contrast material in a region of interest
MTT	mean transit time	Average persistence of a contrast material in a region of interest
AUC	area under the curve	Integral calculus of the area under time-intensity curve, which reflects the total perfusion of a region of interest
BF	blood flow coefficient	BF = AUC/MTT/2

### Histological study (necrosis, proliferation, hypoxia, and microvessel and pericyte density) in HCCLM3 models

Thirty minutes after intraperitoneal injection with 60 mg/kg pimonidazole hydrochloride (Chemicon International, Temecula, CA) [33,34], animals were anesthetized and infused via cardiac puncture with warm PBS followed by 4% paraformaldehyde/PBS. Tumors were dissected with the mark of scanning slice and direction on CEUS and fixed with 4% formaldehyde. The specimens were then dehydrated, embedded in paraffin, and sectioned at 6 μm. Sections were stained with H&E or were subjected to immunohistochemistry study, with primary antibodies against pimonidazole hydrochloride for hypoxia, CD31 (Abcam, Cambridge, MA) for tumor microvessels, and Ki-67 (Dako, Carpinteria, CA) for cell proliferation. Negative controls were treated identically but with the primary antibodies omitted. The components of the Envision-Plus detection system (EnVision/HRP/Mo, Dako) were then applied. Reaction products were visualized by incubation with 3,3-diaminobenzidine. The necrosis index or hypoxia index was calculated as the ratio of necrotic area or pimonidazole stained area to the total area in the whole transverse section. MVD was determined by scanning stained tumor sections at low power, and areas of greatest CD31 positive density were chosen for analysis. The ratio of the positively stained cells to the total area of the field at the magnification of 200× was counted as MVD using Leica QWin Plus v3 software (Leica Microsystems Imaging Solutions, Cambridge, UK), as described previously [10]. The proliferation rate was counted as the proportion of the Ki-67-stained nuclei to the number of total nuclei in the field. MVD and proliferation rate were evaluated in at least 5 randomized chosen fields without necrosis within each tumor. Intratumoral pericyte density was evaluated in frozen sections by immunofluorescence staining with primary antibody against mouse α-smooth muscle actin (α-SMA; Abcam) and Cy5-conjugated anti-rabbit IgG (Jackson ImmunoResearch, West Grove, PA) was used as the second antibody. α-SMA-positive pericytes were counted in the graphics acquired at the magnification of 100× using an inverted fluorescence microscope. α-SMA-positive tumor encapsulation was used as a positive control, but was not calculated as pericytes when processing with Leica QWin Plus v3 software.

### Statistical Analysis

Statistical comparisons were performed using the Student's *t *test when data were normally distributed or using the nonparametric analyses of Spearman and Mann-Whitney when data were not normally distributed. One-way analysis of variance (ANOVA) was used to study the temporal variations of the CEUS parameters during tumor progression or during pazopanib treatment, and the Least Significant Difference test was used to compare between two time points, when necessary. Spearman's rank order correlation was used to investigate the relationships between tumor perfusion and histological features. Two-sided *P *values less than 0.05 were considered statistically significant.

## Results

### In vitro Studies

**Figure 1:**
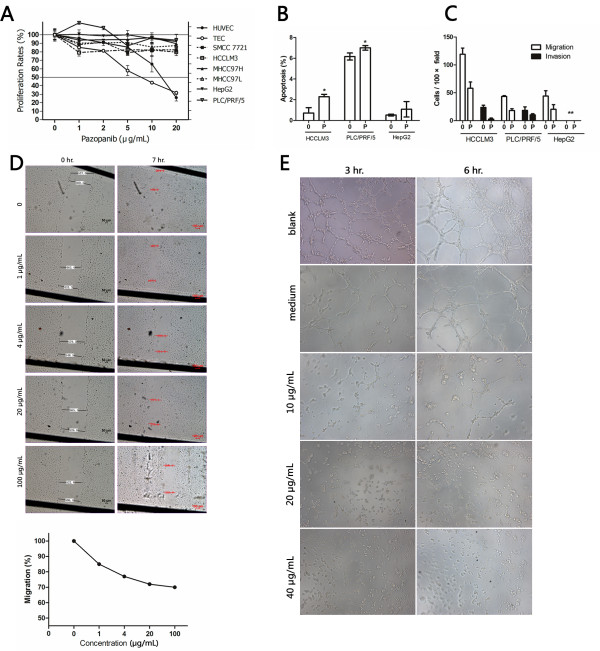
**In vitro effects of pazopanib on hepatoma cells and endothelial cells**. The effects of pazopanib on the in vitro proliferation of various human liver cancer cells, human umbilical vein endothelial cells (HUVEC) and tumor-derived endothelial cells (TEC) at various concentrations (A), and on the apoptosis (B) and migration and invasion of 3 liver cell lines (C) at the concentration of 25 μg/mL. Tubule formation (D) and migration of HUVEC (E) were inhibited by pazopanib in a dose-dependent manner. Each experiment was repeated at least three times. Results of a representative study. Bars, SD; asterisks, *P *< 0.05 as compared with control.

The proliferation and survival of the tested human hepatoma cell lines were slightly affected by pazopanib at concentrations between 1 and 20 μg/mL, whereas the proliferation of HUVEC and TEC was inhibited in a dose-dependent manner (Figure 1A), with an IC_50 _ranging between 5 and 15 μg/mL in 72 h. Pazopanib could induce moderate apoptosis of HCCLM3 and PLC/PRF/5 at the concentration of 25 μg/mL, whereas the apoptosis of HepG2 cells was also not significantly enhanced by pazopanib (Figure 1B). Migration through the filter membrane of the Transwell chamber was significantly inhibited by pazopanib at the concentration of 25 μg/mL in HCCLM3, PLC/PRF/5, and HepG2 cells. The invasion through Matrigel could be also inhibited by pazopanib in these hepatoma cells except in HepG2 cells, which could not invade through the Matrigel even when treated with the vehicle only (Figure 1C). The migration of HUVEC was inhibited by pazopanib in a dose-dependent manner as detected by the wound-healing assay (Figure 1D). When the concentration reached 20 μg/mL, the migration of the HUVEC was reduced by 30% compared with the vehicle-treated cells. Overwhelming cell death could be observed at the concentration of 100 μg/mL, and the migration was difficult to be evaluated. Tubule formation of HUVEC was also inhibited in a dose-dependent manner by incubation with pazopanib for 3 or 6 h. No tube was formed when the concentration reached 20 μg/mL or higher (Figure 1E).

### Pazopanib inhibits xenograft tumor growth

**Figure 2:**
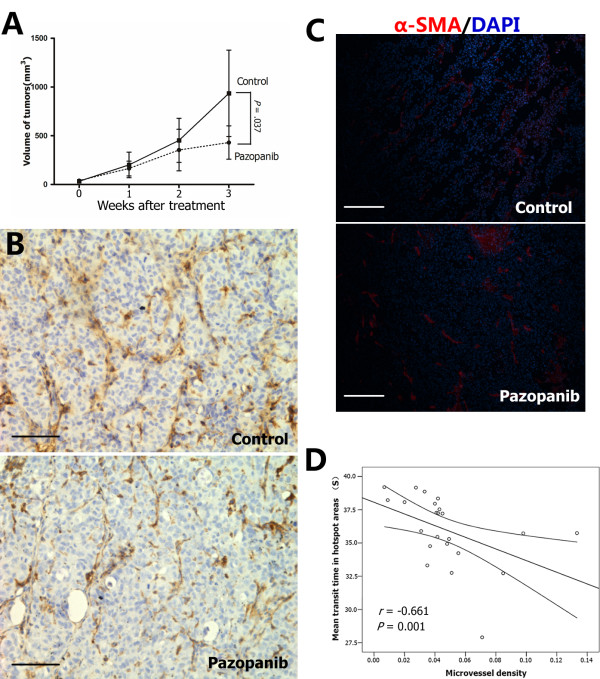
**In vivo effects of pazopanib on the HCCLM3 xenograft model**. Pazopanib inhibited in vivo tumor growth and intratumoral angiogenesis in a subcutaneously implanted HCCLM3 model. Three weeks after the treatment, tumor growth was significantly delayed as compared with the control (A). Microvessels evidenced by anti-CD31 immunostaining were lower in the pazopanib-treated tumors than the tumors treated with vehicle only (B). Endothelial cells formed dilated, disorganized, and tortuous networks in the control, while looking straight and regular in pattern in the pazopanib-treated tumors (B). The density of alpha-smooth muscular actin (α-SMA)-positive pericytes were increased by pazopanib treatment (C). The intratumoral microvessel density reversely correlated with the mean transit time of the contrast flowing through the xenograft tumors detected in the hotspot areas by contrast-enhanced ultrasonography (D). Bars, SD.

A pilot study showed that pazopanib (at a dose of 60 mg/kg daily) significantly inhibited HCCLM3 growth and prolonged the survival of tumor-bearing mice (Additional file 1, Figure S1). To catch the perfusion changes in the course of treatment by weekly applied CEUS, instead of the changes merely at the end of treatment, a lower dosage of 40 mg/kg/d was used in this study. After treated with pazopanib for 3 wk, the tumor growth of HCCLM3 was significantly delayed as compared with the control (0.43 ± 0.17 cm^3 ^versus 0.93 ± 0.44 cm^3^, n = 7 and 5 for each group, *P *= 0.037; Figure 2A). It is also noticed that the difference in tumor size (based on the 3 dimensions detected by ultrasonography in live animals) between the two groups was not statistically significant at the end of 1 wk or 2 wk after the initiation of the treatment (0.16 ± 0.08 versus 0.20 ± 0.13 cm^3^, *P *= 0.364; 0.35 ± 0.21 versus 0.45 ± 0.23 cm^3^, *P *= 0.673; respectively). Tumor growth were also significantly inhibited by 4-week pazopanib treatment in two other xenograft hepatoma models, including orthotopic HepG2 model (0.64 ± 0.16 cm^3 ^in control [n = 6] versus 0.16 ± 0.05 cm^3 ^in pazopanib group [n = 5], *P *= 0.031) and subcutaneous implanted PLC/PRF/5 model (0.86 ± 0.32 cm^3 ^in control versus 0.48 ± 0.11 cm^3 ^in pazopanib group, n = 7 for each group, *P *= 0.012).

### Histological study in HCCLM3 models

At the end of the third week, the mean MVD in the treated tumors was significantly lower than that in the control (3.1% versus 5.8%, *P *= 0.008; Figure 2B). Endothelial cells formed dilated, disorganized, and tortuous networks in the control, while looking straight and regular in pattern in the pazopanib group (Figure 2B). Whereas the intratumoral α-SMA-positive pericytes density in the pazopanib treated tumors was significantly higher than those in control (3.5% ± 0.8% versus 1.4% ± 0.6%, *P *= 0.001; Figure 2C). The necrosis and hypoxia indexes, evidenced by H&E staining and pimonidazole immunostaining, were not significantly changed (64% versus 67%, *P *= 0.729, and 34% versus 35%, *P *= 0.857, respectively). The difference between the proliferation rates of the tumor cells in the pazopanib group and the control was also not significant (33.9% versus 28.1%, *P *= 0.188). It should be noted that the tumor necrosis index correlated well with the tumor size (*r *= 0.670, *P *< 0.001). The tumor in the pazopanib-group was smaller in size but have similar necrosis index in compare with the control group, this may be resulted in the potential pro-necrotic effects of pazopanib. Although a significant correlation was also found between the hypoxia index and tumor size (*r *= 0.719, *P *< 0.001), the standardized hypoxia index in the treated tumors was higher than in the untreated groups, but the difference was not significant (0.8 × 10^-3^/mm^3 ^versus 0.4 × 10^-3^/mm^3^, *P *= 0.092).

### Correlations between tumor perfusion and histological features

Putting the two cohorts (the treated and untreated tumors) together, correlations between the histological features (e.g., necrosis and hypoxia index) obtained in the cross sections of the tumors and the parameters obtained by CEUS were evaluated. Two ROI were selected from each tumor, when the sonography was analysed. One ROI was placed to cover the entire tumor section as a whole, the other was located in the most enhanced area in CEUS, which was referred as a "hotspot" area. Terms used to portray the perfusion by quantitative CEUS and their abbreviations and brief introductions were shown in Table 1.

As shown in Table 2, the necrosis and hypoxia indexes were correlated with some of the velocity-related parameters obtained from the whole tumor (RSI and RWO) of entire tumor (*P *< 0.05). No significant correlation was found between volume-related parameters except ISI, obtained from the entire tumor area and the necrosis index. No significant correlation was found between MVD and all the parameters of CEUS in the entire tumor (Table 1). The correlations between the CEUS parameters from ROIs in the hotspot area and the histological features obtained from non-necrotic areas were evaluated as well (Additional file 1, Table S1). MVD was reversely correlated with MTT measured in the hotspot area (*r *= -0.661, *P *= 0.001; Figure 2D) in the two cohorts (including the treated and untreated tumors, n = 12 in total). And this correlation was true in the treated group (*r *= -0.556, *P *= 0.039, n = 7) but not in the control (*r *= -0.367, *P *= 0.332, n = 5). No statistically significant correlation was found between other histological features (necrosis and hypoxia indexes) or tumor size and all perfusion parameters detected in the hotspot area, except a borderline significant correlation between the necrosis index and BF detected in the hotspot area (*P *= 0.043; Additional file 1, Table S1).

**Table 2:** The correlations between the perfusion parameters measured by contrast-enhanced ultrasonography in the entire transverse tumor area and histological features (n = 12)

		Velocity-related parameters			Volume-related parameters		
			
		RWO	RSI	MTT	ISI	AUC	BF
Necrosis index	*r*	-0.423	-0.656	0.139	-0.482	0.156	0.096
	*P*	0.028	0.000	0.491	0.011	0.436	0.634
Hypoxia index	*r*	-0.470	-0.656	0.08	-0.332	0.269	0.212
	*P*	0.024	0.001	0.72	0.122	0.215	0.330
Microvessel density	*r*	-0.212	0.002	-0.331	0.154	0.318	0.323
	*P*	0.287	0.990	0.092	0.444	0.106	0.100

**Figure 3:**
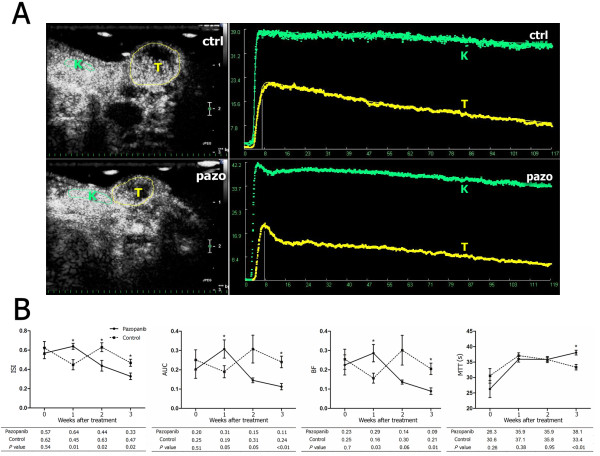
**Intratumoral blood perfusion detected by quantitative contrast-enhanced ultrasonography in pazopanib-treated HCCLM3 xenograft model**. Panel A shows typical images of the contrast-enhanced ultrasonography (CEUS) in subcutaneous tumor (T) and ipsilateral kidney (K) from the control (ctrl) and pazopanib-treated mice (pazo) 3 wk after the initiation of the treatment (left panels). ROIs were selected in the tumor ("T," yellow lines) and ipsilateral kidney ("K," green lines), and the time-intensity curves (right panels) were then obtained in the two regions of interest for further analyses. Tumor perfusion changed in a biphasic manner (panel B). The standardized volume-related parameters detected by CEUS in the whole tumor changed over time. As compared to the control, an increase in signal intensity, the area under the curve, and the blood flow coefficient increased 1 wk after the pazopanib treatment, but decreased afterward. Mean transit time of the contrast in the tumor was also significantly prolonged at the end of the third week of the pazopanib treatment (A). Bars, SEM. Asterisks indicate *P *< 0.05.

Figure 3A showed two typical sonograms and the TICs obtained from the kidney and entire tumor in the mice from the treated group and the control group at the end of the third week of the intervention, when intratumoral blood perfusion was depressed in the treated mice, whereas the blood perfusion of the ipsilateral kidney was slightly affected by pazopanib treatment (Additional file 1, Table S2). Figure 3B presented the perfusion changes of entire tumors in treated and control groups over time. Compared with the control, the volume-related parameters (ISI, AUC, and BF) of the entire tumor in treated tumors were significantly increased at the end of the first week. But at the end of the second week, when the tumor of the treated group was slightly smaller than that of the control (Figure 3B), all these parameters decreased, among which ISI was significantly lowered (Figure 3B). At the end of the third week, the tumor growth was significantly retarded in the treated group and all these parameters were significantly lowered in the pazopanib treated group (Figure 3B). On the other hand, the velocity-related parameters (RSI, RWO and MTT) in treated group changed gradually and were significant at the end of third week (Additional file 1, Table S3), especially the parameter of MTT, which was significantly prolonged at the end of the third week in the treated tumors (Figure 3B). These results indicated that 3-wk pazopanib treatment could lower the peak and the total perfusion of the tumor and could prolong MTT of the contrast material flowing through the tumor.

## Discussion

In this study, we demonstrated the antiangiogenic effects of pazopanib in several human HCC xenograft models, which can be continuously and noninvasively assessed by quantitative CEUS. Oral administration of pazopanib at doses ranging from 10 to 100 mg/kg daily was previously shown to inhibit tumor growth in several human tumor xenograft models [28,35]. The effect of pazopanib on HCC, one of the most vascularized tumors, has not been reported before. Daily treatment with pazopanib at a dose of 40 mg/kg can effectively inhibit tumor growth of 3 different human HCC xenograft models and prolong the life span of tumor-bearing nude mice, which may attribute to the antiangiogenic effects of pazopanib. It remain controversy that tumor cells or endothelial cells, which cell type is main target of the tyrosine kinase inhibitors. In this study, direct anti-proliferation effect of pazopanib on hepatoma cells was not significant either in vitro or in vivo.. Although in vitro pazopanib treatment could induce cell apoptosis and inhibit invasion and migration features in some tumor cells, the concentration of pazopanib is relatively high. To the contrary, we found pazopanib could inhibit the proliferation, migration, and tubule formation of HUVEC in vitro and could reduce the intratumoral MVD in vivo. So, the in vivo antitumor effects of pazopanib may be indirect and may attribute to its antiangiogenic effects, at least at the dosage of 40 mg/kg/d. Similar indirect antitumor effects were reported by Kumar et al. [28] in several other tumor models. Podar et al. [35] found that pazopanib can block multiple myeloma cell growth and survival in vitro. It may therefore be speculated that direct anti-proliferation effects of pazopanib on tumor cells may vary among cell lines.

Using a mathematic model to describe the TIC may reduce the subjectivity of ultrasonography examinations. In the present study, serial parameters were obtained and could reflect the changes of tumor perfusion from different aspects (Table 1), from which ISI stood out. When the efficacy of pazopanib treatment was not proved by tumor size at the end of the second week (Figure 2A), the intratumoral peak perfusion (ISI) detected in the whole tumor started to drop and remained lower afterward, as compared with the control (Figure 3B). Meanwhile, the present study also showed ISI reversely correlated with tumor necrotic area, which was also increased in the treated tumor compared with the untreated tumor. This indicates that the therapeutic response of pazopanib could be sensitively detected by CEUS before a significant change in tumor size, and thus CEUS may possibly meet the demand for a novel method to define the biological response to targeted therapy, which usually did not reduce tumor size and therefore was not appropriate to be judged in traditional ways. The Choi criteria, which evaluated intratumoral necrosis by CT, has been proposed recently [36]. In the present study, several parameters, including ISI obtained from the entire tumor, were found to be significantly correlated with the necrosis index. The necrotic tissue without perfusion reduced the average level of perfusion detected in the entire tumor, and therefore ISI obtained from the whole tumor reflects the mean level of the intratumoral peak perfusion and is affected by the necrosis index. ISI may be used as a comparable parameter in the Choi criteria in CEUS settings. However, ISI also reflects tumor perfusion in the nonnecrotic area, and seems better to compare with the evaluation of the necrosis area alone. The hypothesis of transient "normalizing tumor vasculature" following antiangiogenic therapy [37], whereby blood perfusion within an individual vessel, demonstrated as ISI, AUC, and BF in CEUS, may increase at the end of the first week (Figure 3B).

Another parameter, MTT, obtained from the ROI within the hotspot areas, was also highlighted. MTT obtained from these specific areas was the only parameter found to be associated with the MVD measured in the viable area (Figure 2D). MTT acquired from the entire tumor was also significantly prolonged at the third week in this study (Figure 3B). Although volume-related parameters (e.g., ISI) detected by CEUS were found to be associated with MVD by previous studies [19,38,39], this correlation was not established in this study, probably due to the small sample size and different xenograft models. When microvessels in the viable areas lowered after antiangiogenic therapy, and then the flow-in and wash-out of the contrast was restricted, as a result, MTT, a measurement of the average persistence of a contrast material in the circulation [31], was prolonged. The biologic significance of MTT detected by CT perfusion scan was also revealed in a recently published study; its baseline and change following bevacizumab administration correlated with clinical outcome, whereas the volume parameters (e.g., blood flow and blood volume) did not [14]. When this correlation was further tested in subgroups, we found that the correlation was only established in the treated group. The immature and inefficient neovasculature in the tumor was more susceptible to the VEGF targeting therapy and could be pruned by eliminating excess endothelial cells, and the resulting vasculature is more functional [37]. In this study, the findings that endothelial cells looked straight and regular in pattern and that density of α-SMA-positive pericytes was increased in the tumors from pazopanib-treated mice indicated that vessel normalization may be induced. And as an imaging technology capable of measuring the functional perfusion of contrast media, CEUS can only detect the functional vessels but not the new angiogenic sprouts which are always functionally invalid (not perfused by contrast media). And the new angiogenic sprouts were pruned by pazopanib and were dense in the control (Figure 2B). Further histological studies are needed to define the morphological and functional differences of the microvessels between the two groups and explain why the correlation between MTT and MVD could be found only in the treated group. More recently, Palmowski et al [40] evaluated large vessels and immature vessels with high-frequency Doppler ultrasound technique equipped by VisualSonics. Compared with the very high-frequency transducer which is only eligible for small animals, clinically available transducer used in the study had relatively lower resolution for tumor vessels. Although attempted, the CEUS system we used in this study could detect the blood perfusion of the subcutaneous tumors but not of the intrahepatic ones, therefore intratumoral perfusion of orthotopic HepG2 tumors were not detected in this study. However, the quantitative assessment of intratumoral perfusion is another method to detect the functional vessels in tumors treated with antiangiogenesis. The present study provides a rationale for testing the therapeutic effects of pazopanib or other targeted agents with antiangiogenic effects in patients with unresectable HCC.

This study has some limitations. First, although in vivo effects of pazopanib were evaluated in 3 human HCC nude mice models, other dosage and schedules of pazopanib or spontaneous model of liver cancer were not tested in this study. Second, fine changes of tumor perfusion may not be observed and only the subcutaneous tumor models could be detected by this equipment due to its relatively lower resolution and small tumor size. In the clinical settings, the tumors are always of large size, especially in the HCC patients with unresectable tumors which are the indications of antiangiogenic therapy. CEUS examination should be practical in these patients. Third, the burst/replenishment kinetics which had some advantages in minimizing the influence of cardiac output [41] was not used in our study. We took the perfusion of contrast in the ipsilateral kidney as a control for instead, which may also reduce the influence of cardiac output.

## Conclusions

In humans, pazopanib is currently being studied in more than 40 clinical trials in various cancers, including a phase I study to evaluate the safety, tolerability, and optimal dosage in HCC patients. The findings of our study provide a rationale for testing the therapeutic effects of pazopanib in patients with unresectable HCC. As CEUS has several advantages over DCE-MRI and DCE-CT [42], the application of quantitative CEUS, especially the two parameters ISI and MTT, deserves to be further evaluated in comparative clinical trials, especially in much larger unresectable tumors.

## List of abbreviations used

HCC: hepatocellular carcinoma; CEUS: contrast-enhanced ultrasonography; VEGF: vascular endothelial growth factor; VEGFR: VEGF receptor; MVD: microvessel density; HUVEC: human umbilical vein endothelial cells; DCE-CT: dynamic contrast-enhanced computed tomography; DCE-MRI: dynamic contrast-enhanced magnetic resonance imaging; DMSO: dimethyl sulphoxide; ANOVA: analysis of variance; TIC: time-intensity curve; ROI: region of interest; ISI: increased signal intensity; RSI: rate of signal increase; RWO: rate of washout of 50% contrast material; MTT: mean transit time; AUC: area under the curve; BF: blood flow coefficient.

## Competing interests

The authors declare that they have no competing interests.

## Authors' contributions

HD and HCS participated in the design of the experiments, data interpretation, and manuscript preparing. XDZ, JBZ, and PLF equally contributed to this work. They performed the experiments, including in vitro experiments and histological studies (XDZ and JBZ) and in vivo experiments (PLF), analyzed the data, and participated in writing of the manuscript. Ultrasonography was carried out by PLF and HD. YQX, PYZ, WZ, HXX, DMG, and LQK participated in the in vivo studies and histological studies. LW, WZW, and ZYT participated in the design of the experiments. All authors read and approved the final manuscript.

## Pre-publication history

The pre-publication history for this paper can be accessed here:

http://www.biomedcentral.com/1471-2407/11/28/prepub

## Supplementary Material

Additional file 1 **Additional results including 2 figures and 3 tables**. Two figures (Figure S1-S2) and 3 tables (Table S1-S3) were included in this file. Click here for file
