# Supplementary material for: Antiangiogenic effects of pazopanib in xenograft hepatocellular carcinoma models: evaluation by quantitative contrast-enhanced ultrasonography
Source: BMC Cancer. 2011 Jan 20;11:28. doi: 10.1186/1471-2407-11-28 (PMC3033852; doi:10.1186/1471-2407-11-28)
Supplement: Additional file 1 — Additional results including 2 figures and 3 tables. Two figures (Figure S1-S2) and 3 tables (Table S1-S3) were included in this file. [file 1471-2407-11-28-S1.DOC]

# Additional files

Two figures and 3 tables were included.

## Additional Figures

## Figure S1 - Pazopanib shows the therapeutic effects in a pilot study

A pilot study was performed to determine the optimal dose of pazopanib. Pazopanib was administrated 60 mg/kg daily by oral gavage, tumor volume was measured every other day, and survival time was evaluated from the first day of treatment until death. By significantly delaying the tumor growth of the xenograft tumor (n = 12 for each group), the survival time of the tumor-bearing mice was significantly prolonged by oral pazopanib (median survival time, >50 d in pazopanib-treated group versus 42.6 d in the control, *P* = 0.021). Bars, SD.

## Figure S2 - Schematic of the time intensity curve obtained from a region of interest detected by contrast-enhanced ultrasonography

ISI, increased signal intensity from baseline to the peak enhancement; RSI, rate of signal increase; RWO, rate of washout of 50% contrast material; AUC, area under the curve.

## Figure S1

**A**

**B**

## Figure S2


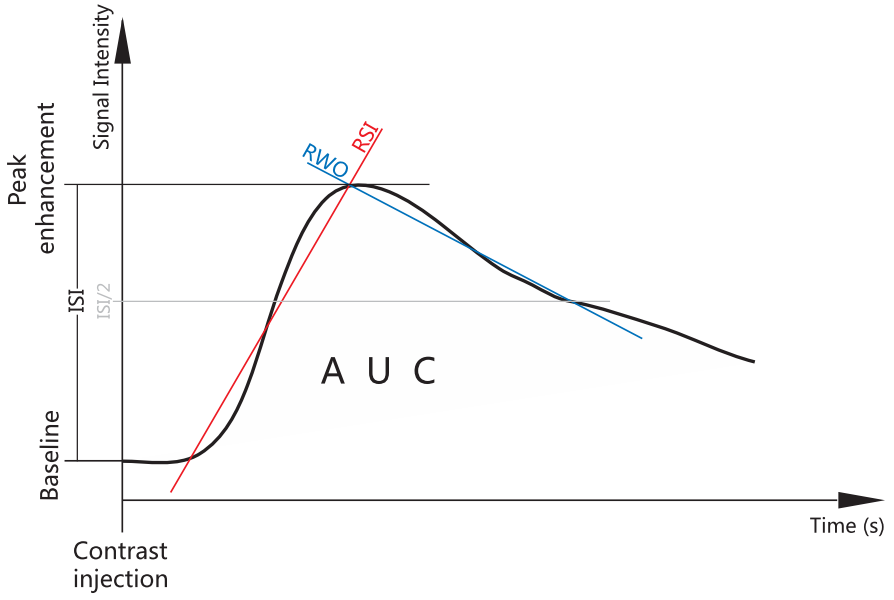


## Additional Tables

## Table S1 - The correlations between perfusion parameters detected by contrast-enhanced ultrasonography in the hotspot areas and histological features (n = 12)

|  |  | Velocity-related parameters | | |  | Volume-related parameters | | |
| --- | --- | --- | --- | --- | --- | --- | --- | --- |
|  |  | RWO | RSI | MTT |  | ISI | AUC | BF |
| Necrosis index | *r* | -0.30 | -0.06 | -0.04 |  | -0.27 | -0.39 | -0.42 |
|  | *P* | 0.16 | 0.79 | 0.86 |  | 0.21 | 0.06 | 0.04 |
| Hypoxia index | *r* | -0.37 | -0.32 | 0.02 |  | -0.14 | -0.16 | -0.21 |
|  | *P* | 0.10 | 0.16 | 0.94 |  | 0.54 | 0.49 | 0.35 |
| Microvessel density | *r* | -0.28 | -0.04 | -0.66 |  | 0.07 | 0.16 | 0.16 |
|  | *P* | 0.19 | 0.87 | <0.01 |  | 0.75 | 0.48 | 0.47 |

## Table S2 - The volume-related perfusion parameters of the right kidney detected by contrast-enhanced ultrasonography at the end of each week after the initiation of treatment

| Week | ISI | | |  | AUC | | |  | BF | | |
| --- | --- | --- | --- | --- | --- | --- | --- | --- | --- | --- | --- |
|  | pazo | ctrl | *P* |  | pazo | ctrl | *P* |  | pazo | ctrl | *P* |
| 0 | 32.4 | 34.2 | 0.50 |  | 4544 | 4595 | 0.95 |  | 78.9 | 76.0 | 0.86 |
| 1 | 38.8 | 34.3 | 0.02 |  | 5410 | 6055 | 0.59 |  | 86.0 | 102.3 | 0.49 |
| 2 | 31.8 | 35.5 | 0.07 |  | 3791 | 4146 | 0.60 |  | 56.3 | 64.3 | 0.53 |
| 3 | 38.8 | 38.1 | 0.76 |  | 8608 | 10608 | 0.10 |  | 140 | 187.8 | 0.05 |

Note. pazo, pazopanib; ctrl, control.

## Table S3 - The quantitative parameters detected in the whole tumor by contrast-enhanced ultrasonography at the end of each week after the initiation of treatment (n = 12)

| Week | RWO | | |  | RSI | | |  | MTT (s) | | |
| --- | --- | --- | --- | --- | --- | --- | --- | --- | --- | --- | --- |
|  | pazo | ctrl | *P* |  | pazo | ctrl | *P* |  | pazo | ctrl | *P* |
| 0 | 0.068 | 0.034 | 0.11 |  | 2.84 | 3.17 | 0.40 |  | 26.3 | 30.6 | 0.26 |
| 1 | 0.018 | 0.018 | 0.98 |  | 3.74 | 1.83 | <0.01 |  | 35.9 | 37.1 | 0.38 |
| 2 | 0.023 | 0.022 | 0.06 |  | 2.66 | 3.17 | 0.37 |  | 35.9 | 35.8 | 0.95 |
| 3 | 0.016 | 0.007 | <0.01 |  | 2.17 | 1.17 | 0.02 |  | 38.1 | 33.4 | <0.01 |

Note. pazo, pazopanib; ctrl, control.
